# The selfish germ

**DOI:** 10.1371/journal.pbio.2003250

**Published:** 2017-07-12

**Authors:** Andrew F. Read

**Affiliations:** Center for Infectious Disease Dynamics, Departments of Biology and Entomology, Penn State, University Park, Pennsylvania, United States of America

## Abstract

Curiosity about the sex life of a wasp led to a new way of thinking and a powerful demonstration that evolutionary science could be predictive. That same approach could help find ways to slow or prevent treatment failures in cancer and infectious diseases.

Sometime in the early 1960s, a graduate student read “with near incredulity” a 1922 paper about a tiny wasp. The wasps were fully sexual but produced almost no males. Back then, expert opinion was that natural selection favored 1:1 sex ratios. The student, W. D. Hamilton, was so puzzled that he started a file called “The *Melittobia* Problem,” named for the insect. [1]. Several years later, he published the answer. It was incest. *Melittobia* females mate with their brothers and their sons. Simple calculus led Hamilton to predict for any organism that the more inbreeding mothers do, the fewer sons they will produce [2]. That was something unheard of in biology: a novel, inescapably quantitative prediction of staggering generality. Field naturalists went looking. It turned out he was right [3].

What Hamilton had done was to reason mathematically about how natural selection must work in the environment in which organisms are actually living. That way of thinking, selection thinking, was later explained by Richard Dawkins in *The Selfish Gene*. To understand trait evolution, you have to compare the survival and reproduction consequences of the possible states that a trait could take. It seems obvious now, and a few were thinking that way back then, but they were mostly doing it without mathematics. Hamilton showed that relatively simple calculations could get you both rigor and prediction. I well remember discovering as a graduate student the sheer and utter beauty of it all. Towards the end of my PhD, I had to try it myself. If Hamilton’s sex ratio theory was that good, it should apply to my new interest, malaria parasites.

Take a quick look at **Fig 1**. The details aren’t important. Absent Hamilton’s theory, there is no reason to even consider drawing such a plot. But after we had done a little algebra, it was clear that I would be doing just that because, radically, Hamilton’s theory predicted that malaria parasite populations should not be all over that space or even clustered around 1:1 but, instead, that evolution should have put them in the small region between the blue lines. The first thing I did when I set up my own lab in the early 1990s was test that prediction by sampling sex ratios in parasite populations from around the world. I still recall the spine tingles each time new data came in. Within measurement error, every one of the populations fell into the zone [4]. Wow. The solution to a problem identified in an obscure wasp applied to microbes that were then killing over a million people a year. Wow. Hamilton’s theory, it turned out, was that good.

**Fig 1:**
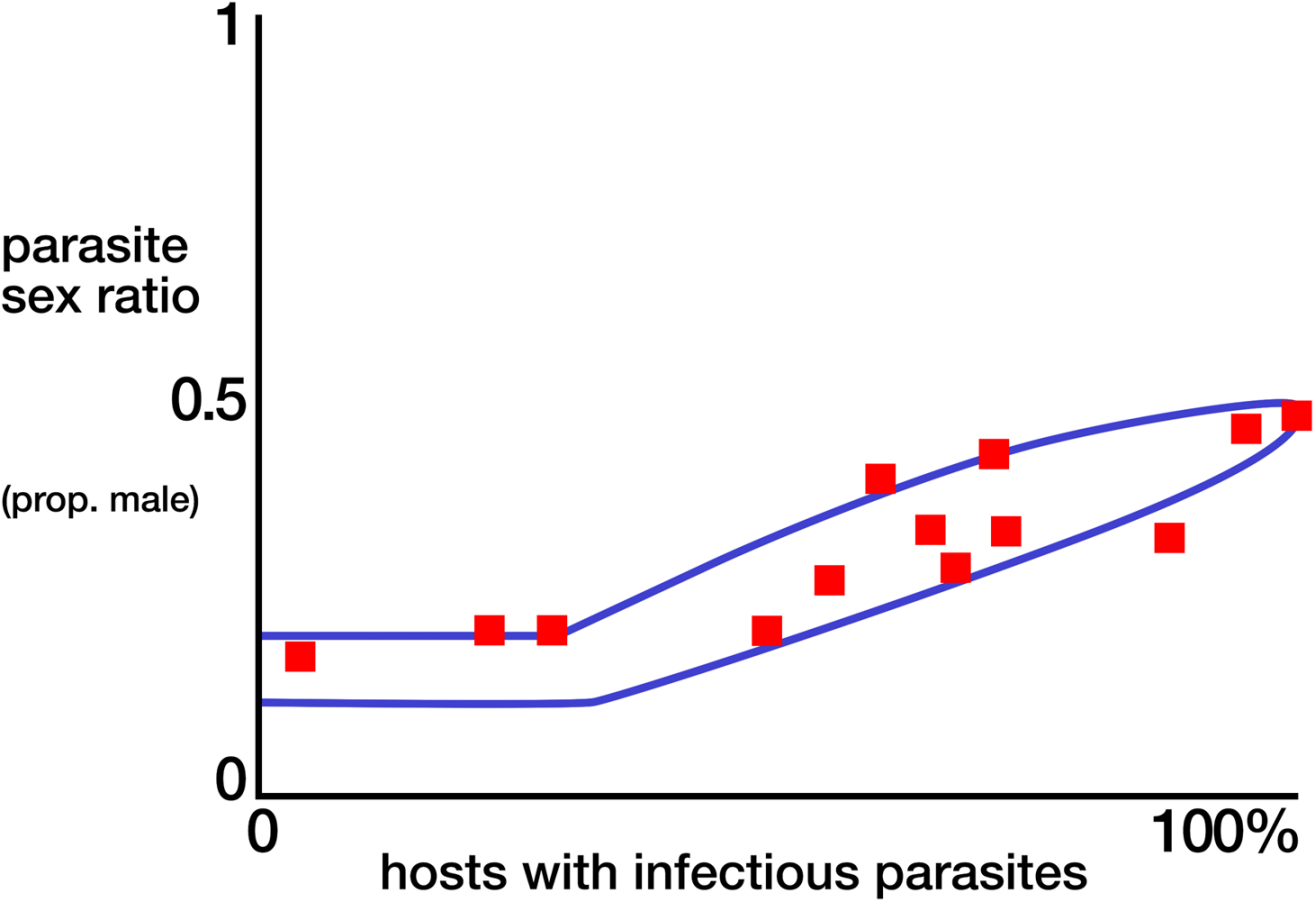
Prevalence and sex ratios of the transmission stages of malaria parasites in the peripheral blood of vertebrate hosts in various populations (red points). The theoretical expectation lies within the blue lines. For details, see [4].

Reviewers were unimpressed. As they were at pains to point out, Hamilton’s theory was by then well verified and nobody really cares about malaria sex ratios (they don’t make you sick). But I was stunned: selection thinking had empowered me to make a novel prediction that checked out. That’s how grown-up science was supposed to work—and I could do it!

From that success came the confidence to bet my career on the next step: applying selection thinking to more complex microbial traits, not least those that do make people sick. And that’s been very rewarding because it has let me see things I never expected. One was the realization that some vaccines can enable more virulent pathogens to evolve [5]. That claim proved highly controversial, but it takes just a few lines of Hamiltonian-like calculus to get there from the widely accepted view that pathogens that are too nasty have no future. Some years after we did the calculus, our experiments in lab and farm animals provided proof of principle [6,7].

Another thing I got to see was that it is possible to make insecticides for malaria control that will never be undermined by insecticide resistance. An early success of selection thinking to which Hamilton also made seminal contributions [1] was the insight that natural selection does not favor life-enhancing genes if they only act late in life. That explains why we age. It also means that insecticides that only act on older mosquitoes, the ones that transmit malaria, cannot fail even when insecticide-resistance genes are present [8]. There is no selection to survive insecticide resistance if death will happen anyway.

The prospect of evolution-proof insecticides got me into a third area, one that has yet to fully work itself out. Evolution kills about 600,000 people each year in America alone. Cancers have a ferocious capacity to evolve themselves around the insults oncologists throw at them, and an increasing number of people die from infections that were readily treated with antibiotics when I was a graduate student. Could selection thinking suggest ways to slow or redirect that life-threatening resistance evolution? A growing number of us think so [9–16].

Consider, for instance, the following challenge. How should we treat patients when resistance to medications is already present in a tumor or infection and there are no other options? Standard practice is to aggressively treat infections and cancers to stop them becoming resistant. But that notion always troubled me. Drug use causes drug resistance. So why keep taking drugs when the patient no longer feels sick, especially when the drugs themselves sicken? For sure, killing cancer cells or infectious agents stops them becoming resistant (dead things don’t mutate). But a firestorm of drugs removes the competitors of the very things we fear: the cells and bugs we can’t kill. Expert opinion is that stopping mutations is more important than competition, and if that’s right, today’s standard practice is indeed best. But where it is wrong, less aggressive regimens may be better [15]. There are circumstances in which patients will live longer if drugs are used to contain life-threatening tumors and chronic infections rather than try to cure them. Selection thinking makes it possible to define those situations [16].

Could selection thinking really prolong lives? The math is harder for therapy resistance than for sex ratios, but the fundamentals are the same, and so it should work. Selection thinking is by no means the only way to try to head off the real-time evolution that harms human well-being, but it is one way. And in no small part, we have it because a graduate student got curious about the sex life of a little wasp.
